# Latent profile analysis of Eysenck’s personality dimensions and psychological constructs in university students

**DOI:** 10.3389/fpsyg.2024.1379705

**Published:** 2024-05-09

**Authors:** Lin-Ling Pan, Si-Ran Zhou, Guan-Zhao Chen, Yue-Dan Ke, Zi-Ye Huang, Yu-Wei Wu, Wen-Jing Yan

**Affiliations:** ^1^Wenzhou Seventh People’s Hospital, Wenzhou, China; ^2^School of mental health, Wenzhou Medical University, Wenzhou, China; ^3^Graduate School, University of the East, Manila, Philippines; ^4^Student Affairs Division, Wenzhou Business College, Wenzhou, China; ^5^Zhejiang Provincial Clinical Research Centre for Mental Illness, Affiliated Kangning Hospital, Wenzhou Medical University, Wenzhou, China

**Keywords:** Eysenck’s personality traits, latent profile analysis, psychological constructs, university students, anxiety

## Abstract

**Background:**

The exploration of personality traits in relation to psychological constructs has become increasingly relevant in understanding the mental health of university students (the emerging adulthood). Studies have focused on how dimensions intersect with various psychological parameters.

**Aim:**

The study aims to identify distinct personality profiles among university students based on Eysenck’s personality dimensions and investigate how these profiles differ across psychological constructs.

**Method:**

A quantitative methodology was utilized, involving 708 university students from Wenzhou and Nanjing in China as participants. The research employed the Eysenck Personality Questionnaire along with other psychological measures. Latent Profile Analysis was applied to categorize the participants into distinct personality profiles.

**Results:**

Four distinct personality profiles emerged: ‘The Reserved Analyst,’ ‘The Social Diplomat,’ ‘The Unconventional Pragmatist,’ and ‘The Impulsive Truth-Teller.’ Significant differences were found among these profiles on various psychological constructs. ‘The Social Diplomat’ exhibited the most adaptive psychological profile, with higher cognitive reappraisal (*F* = 45.818, *p* < 0.001, *η*^2^ = 0.163), meaning in life (*F*  = 17.764, *p* < 0.001, *η*^2^ = 0.070), and positive coping (*F* = 40.765, *p* < 0.001, *η*^2^ = 0.148) compared to other profiles. Conversely, ‘The Reserved Analyst’ showed higher intolerance of uncertainty (*F* = 13.854, *p*  < 0.001, *η*^2^ = 0.056) and state anxiety (*F* = 26.279, *p*  < 0.001, *η*^2^ = 0.101).

**Conclusion:**

This study enriches the understanding of personality traits in relation to psychological constructs within the context of university student populations. By identifying distinct personality profiles, it lays the groundwork for developing tailored mental health strategies that cater to the specific needs of different student groups.

## 1 Introduction

In recent years, the intersection of personality dimensions with emotional regulation, coping mechanisms, and mental health has garnered increasing scholarly attention (Barańczuk, 2019; Chervonsky and Hunt, 2019). This surge in interest aligns with a heightened awareness of mental health issues among university students, who are navigating pivotal life transitions and challenges. University students fall within the developmental stage of emerging adulthood, a distinct period between adolescence and young adulthood, typically spanning the ages of 18–25 (Arnett, 2000). This stage is characterized by identity exploration, instability, self-focus, feeling in-between, and possibilities (Arnett, 2023). The unique challenges faced by emerging adults can be better understood by examining the interplay between personality traits, cognition, and emotion within this developmental context.

Focusing on Eysenck’s personality traits—extraversion, neuroticism, and psychoticism (Eysenck, 1983)—this research offers deep insights into how these traits shape emotional and psychological responses in emerging adults. By correlating these traits with other psychological constructs like emotion regulation, coping styles, and anxiety levels, we enhance our understanding of the complex psychological dynamics involved in this specific population. The key features of emerging adulthood, such as identity formation, increased autonomy, and shifting social roles, can interact with personality traits to shape psychological well-being (Schwartz et al., 2013). For example, high neuroticism may exacerbate the stress and uncertainty that often characterize this period, while extraversion may facilitate the formation of supportive social networks. This integrative approach not only illuminates the interplay between personality, cognition, and emotion but also advances our comprehension of university students’ psychological health within the context of emerging adulthood. Such a developmentally-informed understanding is instrumental in devising more tailored and effective mental health interventions for this demographic. By framing our study within the emerging adulthood model, we aim to offer a more nuanced interpretation of our findings and their implications for supporting university students’ mental health during this critical stage of development.

### 1.1 Personality traits and psychological constructs

The existing body of research on personality traits and psychological constructs in university students offers a rich tapestry of findings, yet it also reveals areas needing further exploration. Central to this discussion is the Eysenck Personality Questionnaire (EPQ) (Rocklin and Revelle, 1981; Barrett et al., 1998), a widely used measure of personality traits, including extraversion, neuroticism, and psychoticism. Eysenck’s model has been a cornerstone in personality research, providing valuable insights into how these traits influence various psychological outcomes.

Studies leveraging the EPQ have consistently demonstrated the significant role of extraversion in predicting positive psychological outcomes (Klinger-König et al., 2018). Extraverts, characterized by their sociability and assertiveness, often exhibit better stress management and coping strategies (Vollrath and Torgersen, 2000). This is supported by a study (Soto and John, 2017), which found a strong correlation between extraversion and positive affect, suggesting that extraverted individuals are better equipped at managing stress and maintaining positive emotional states.

Conversely, neuroticism, defined by a tendency toward anxiety and emotional instability, has been linked to a range of psychological challenges (Barlow et al., 2014). High neuroticism scores are often associated with poorer mental health outcomes (Lahey, 2009; Kotov et al., 2010). Their study highlighted the relationship between high neuroticism and increased susceptibility to mental health disorders, including anxiety and depression (Ormel et al., 2013; Nikčević et al., 2021).

The dimension of psychoticism, though less frequently explored, has shown intriguing associations with non-conformity and creativity, as well as with antisocial behaviors. A study illuminated the complex role of psychoticism in both adaptive and maladaptive behaviors, suggesting that while it may predispose individuals to challenging behaviors, it could also foster creativity and original thinking (Sampedro et al., 2020).

Furthermore, the interplay between these personality traits and other psychological constructs such as coping styles, anxiety, and sense of life’s meaning has been a focus of recent research. For example, the Positive Psychology framework, which emphasizes personal strengths and well-being, has been instrumental in exploring how personality traits influence coping mechanisms and overall psychological resilience (Wagner et al., 2020; Smith et al., 2021). Studies within this framework have shown that certain personality traits can enhance an individual’s ability to thrive in the face of adversity, promoting a more positive and adaptive psychological outlook.

### 1.2 Research gaps

Several gaps and unexplored areas persist.

First, most existing studies have examined personality traits in isolation, without considering the interactive effects of different personality dimensions on psychological outcomes. This presents a significant gap, as the interaction between traits like extraversion, neuroticism, and psychoticism can offer a more nuanced understanding of their collective impact on psychological well-being (Heinonen and Nissen-Lie, 2020). For instance, the interplay between high extraversion and low neuroticism in relation to coping strategies remains underexplored. Our study seeks to address this by examining how these traits work in concert, particularly in the unique setting of a university environment.

Second, the application of Latent Profile Analysis (LPA) in this context is relatively novel (Spurk et al., 2020). LPA allows for the identification of distinct personality profiles within the population, providing a more sophisticated understanding of how various traits cluster together in real-world settings. Prior research has predominantly used traditional statistical methods, which may not fully capture the complexity and nuances of personality traits. By employing LPA, our study aims to uncover distinct personality profiles within university students, offering a more detailed and practical understanding of their psychological makeup.

### 1.3 The present study

Our study is positioned to address these gaps by exploring the interactive effects of different personality traits, focusing on a specific and critical demographic, employing advanced methodological approaches like LPA and network analysis, and adopting a longitudinal perspective. The objectives of our study are to identify distinct personality profiles among university students, understand how these profiles relate to various psychological constructs, and explore the implications of these relationships for psychological interventions and support mechanisms. This approach not only aims to contribute to the academic discourse but also holds practical significance for the development of tailored mental health strategies for university students.

## 2 Method

### 2.1 Participants

Participants were undergraduate, master’s, and doctoral students from various colleges and universities in Wenzhou and Nanjing, China. A simple random cluster sampling method was employed between 2021 and 2022. The study was conducted online using the Wenjuanxing platform. Before completing the questionnaires, participants were required to read and agree to the consent form. Those who participated in the study were eligible to earn credits. Individuals diagnosed with reading disorders and those who refused to provide informed consent were excluded from the study. To enhance data reliability, a teaching manipulation check was embedded in the assessment to identify and exclude casual or insincere responses. Of the 850 participants who initially began the study, 142 were excluded based on their responses to the teaching manipulation check. The final sample consisted of 708 students (247 men, 34.89%; 461 women, 65.11%) who provided valid and usable data, resulting in a valid response rate of 83.29%.

### 2.2 Measures

#### 2.2.1 Eysenck personality questionnaire

The questionnaire in its Chinese version comprises a total of 88 items, scored dichotomously with gender-based norm divisions (Qian et al., 2000). It is structured into four subscales: Extraversion/Introversion (E), Neuroticism/Stability (N), Psychoticism/Socialization (P), and a validity scale (L). Professor Gong’s revised Chinese version holds a favorable reputation among his Chinese counterparts. Notably, for the adult version, he computed coincidence indicators rather than reliability, revealing a coincidence rate between the items in the revised version and the original questionnaire ranging from 87.5 to 97.82%. In this study, the Cronbach’s α coefficient for the E dimension was 0.788, for the N dimension was 0.872, for the P dimension was 0.697, and for the L dimension was 0.682.

#### 2.2.2 Emotion regulation questionnaire

Emotion regulation questionnaire (ERQ), crafted by James Gross and his team at Stanford University in 2003, encompasses two key aspects: cognitive reappraisal and expressive suppression, spread across a total of 10 items (Preece et al., 2021). A higher score on the questionnaire signifies a more frequent employment of emotion regulation strategies by an individual. Specifically, within these two dimensions, an elevated average score reflects more frequent usage of the respective emotion regulation tactic. The Chinese adaptation of the ERQ has been proven to possess robust reliability and validity. The test–retest reliability and internal consistency for the cognitive reappraisal dimension stand at 0.82 and 0.85, respectively, while those for the expressive suppression dimension are 0.79 and 0.77. In this study, the ERQ demonstrated a Cronbach’s α coefficient of 0.814, with the cognitive reappraisal and expressive suppression dimensions yielding coefficients of 0.833 and 0.727, respectively.

#### 2.2.3 The intolerance of uncertainty scale

The intolerance of uncertainty scale (IUS-12), comprising 12 items, assesses an individual’s comfort with uncertainty (Wilson et al., 2020). This scale employs a 5-point Likert scale, where responses range from “completely disagree” to “completely agree,” and a higher score denotes a lower tolerance for ambiguity. The internal consistency reliability of the overall scale and its specific dimensions varies between 0.704 and 0.878, while the test–retest reliability falls between 0.695 and 0.78. In this study, the Cronbach’s α coefficient for the IUS-12 was reported to be 0.829.

#### 2.2.4 The meaning in life questionnaire

Meaning in life questionnaire (MLQ), created by Steger and colleagues, features 10 items divided into two dimensions (Steger et al., 2006). The Search for Meaning in Life (SML) and the Presence of Meaning in Life (PML). This questionnaire employs a 7-point scale where higher scores reflect a greater perceived sense of life’s meaning, as outlined by Steger et al. in 2006. The Chinese adaptation of the MLQ reported a total Cronbach’s α coefficient of 0.71. In the context of this study, the questionnaire demonstrated a Cronbach’s α coefficient of 0.833.

#### 2.2.5 The simplified coping style questionnaire

Simplified coping style questionnaire (SCSQ) is a self-reported scale with 20 items designed to evaluate an individual’s coping strategies (Xie, 1998). It’s divided into two subscales: positive coping, consisting of 12 items, and negative coping, with 8 items. Positive coping is indicative of a proactive approach, involving strategies like problem-solving through work or learning, and focusing on the positives. Conversely, negative coping is characterized by a more passive approach, such as avoidance through drinking and smoking or depending on others to resolve issues. Responses are measured on a four-point Likert scale, with higher scores on each subscale representing a greater prevalence of that coping style. The Cronbach’s α coefficients for positive and negative coping are 0.89 and 0.78, respectively.

#### 2.2.6 The self-identity scale

Self-identity scale (SIS) was utilized to determine if individuals have navigated through the identity crisis as described by Erickson (Ochse and Plug, 1986). This questionnaire contains 19 items rated on a 4-point scale, where higher scores suggest a well-developing personal identity and lower scores suggest the opposite. The internal consistency coefficient for the Chinese adaptation of the SIS was recorded at 0.727. In the context of this study, the Cronbach’s α coefficient for the questionnaire was 0.785.

#### 2.2.7 State anxiety inventory

The State–Trait Anxiety Inventory (STAI) is a widely recognized tool in psychological assessment, specifically designed for measuring anxiety in adults (Bieling et al., 1998). It is comprised of two distinct self-report scales, of which the State Anxiety Scale (S-AI) is one. This particular scale, comprising 20 items, is focused on evaluating an individual’s feelings of anxiety and stress at a specific moment in time, encapsulating their current emotional state. Each item on the S-AI is rated on a 4-point Likert scale, which ranges from “Not At All” to “Very Much So.” This allows for a nuanced assessment of immediate anxiety levels. The total score for the State Anxiety Scale can vary from 20 to 80, with higher scores reflecting greater immediate anxiety. In the study we used Chinese version (Shek, 1993), the Cronbach’s α coefficient for the State Anxiety Scale was notably high at 0.932, indicating a strong reliability in measuring state anxiety.

### 2.3 Statistics analysis

Data analysis began with the calculation of descriptive statistics. In addition to the conventional correlation matrix, we also employed network analysis, which is quite popular recently to display the relationships among the variables. Following the preliminary analyses, Latent Profile Analysis (LPA) was employed to identify distinct subgroups within the population based on the four EPQ dimension scores used as observational indicators. Aiming to categorize individuals into profiles, the LPA underwent a rigorous model selection process to determine the optimal number of profiles. The fit of the models was assessed using several criteria, including the Akaike Information Criterion (AIC), Bayesian Information Criterion (BIC), adjusted BIC (aBIC), and entropy. Lower values of AIC, BIC, and aBIC indicate a better fit of the model to the data, while entropy, ranging from 0 to 1, reflects the precision of classification within the model, with values closer to 1 suggesting higher accuracy. Entropy values exceeding 0.8 are generally indicative of a model that correctly classifies over 90% of subjects (Celeux and Soromenho, 1996).

To further validate the number of profiles, we evaluated the model using the Lo–Mendell–Rubin Test (LMRT) and the Bootstrapped Likelihood Ratio Test (BLRT). These tests compared the goodness of fit between k-level models and k-1-level models, with significant *p*-values (below 0.05) indicating that models with more levels fit the data better.

Having identified the most appropriate LPA model, the subsequent analysis focused on understanding the differences between the identified latent traits. A one-way analysis of variance (ANOVA) was used to assess differences in continuous scores between traits. These analyses provided insight into the different characteristics and behaviors associated with each trait, leading to a deeper understanding of the underlying patterns in the data.

The analyses were conducted using advanced statistical software, specifically SPSS 26.0 for descriptive and inferential statistics and Mplus 8.3 for the Latent Profile Analysis.

## 3 Result

### 3.1 Descriptive statistics

**Figure 1:**
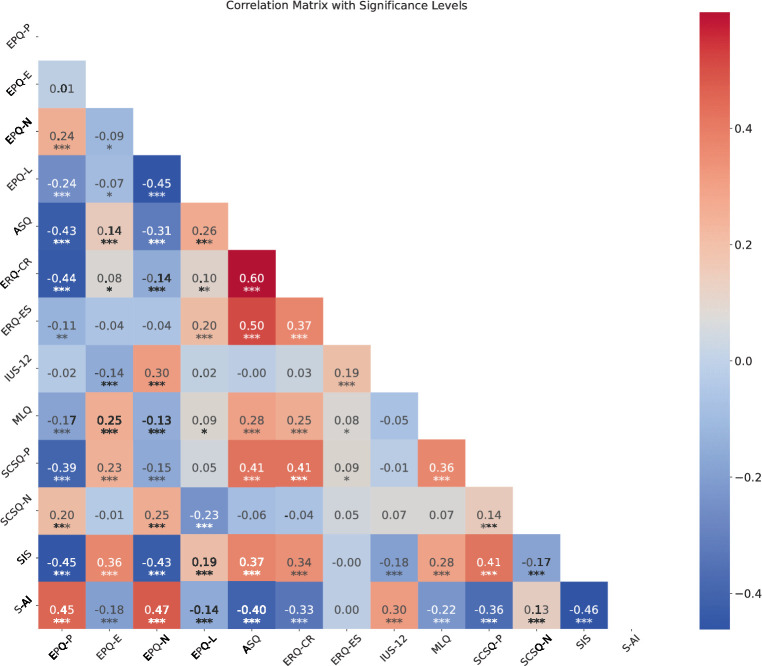
Correlation Heatmap displaying the relationships between various psychological measures with significance levels. Red shades represent positive correlations and blue shades represent negative correlations, with asterisks denoting the significance of each correlation.

**Figure 2:**
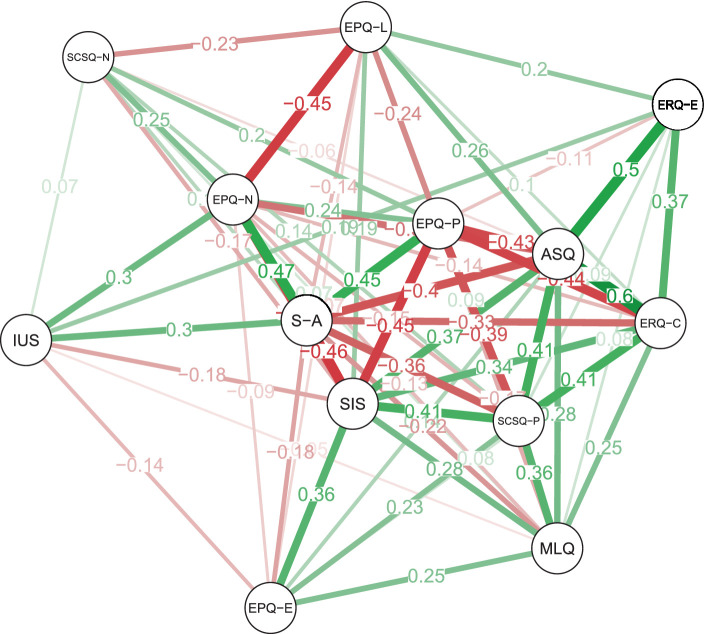
Network analysis of psychological constructs. Nodes represent different psychological measures, with line thickness denoting the strength of correlation, and color indicating the type (green for positive and red for negative correlations).

The sample for this study included 708 participants, of whom 247 (approximately 34.9%) were man. The age of participants was 21.32 (SD = 3.03) years. Correlation analysis revealed some strong associations. Correlation Heatmap displaying the relationships between various psychological measures with significance levels (see Figure 1). In addition, to better display the relationship between these variables we employ the network analysis which can show the complicated relationship among them (Figure 2).

### 3.2 Latent profile analysis

This research applied Latent Profile Analysis (LPA) to delve into the diverse categories within the EPQ. Details of how well each LPA model performed are depicted in Table 1. When comparing the models, the two-class LPA model outperformed the single-class one, as indicated by lower AIC, BIC, and aBIC values and a significant BLR test, suggesting a better fit. This pattern continued, with each additional class improving the model’s fit up until the six-class model, which was an improvement over the five-class model. However, the two, three, and six-class models showed lower Entropy values, hinting at less reliable classification. Given the overlap in the five-class model and the goal for a simpler yet accurate model, the four-class LPA model was chosen as the best fit.

**Table 1:** LPA model fitting information for profiles 1 to 6.

Model	AIC	BIC	aBIC	Entropy	LMRT(p)	BLRT(p)
Profile1	21669.798	21706.297	21680.896			
Profile2	21436.802	21496.114	21454.836	0.627	<0.001	<0.001
Profile3	21354.153	21436.277	21379.123	0.683	<0.001	<0.001
Profile4	21296.436	21401.372	21328.342	0.727	<0.001	<0.001
Profile5	21275.614	21403.362	21314.456	0.720	<0.05	<0.001
Profile6	21245.827	21396.387	21291.605	0.695	0.648	<0.001

The breakdown of the EPQ average scores for each category is shown in Figure 1. We found four profiles according to LPA.

**Figure 3:**
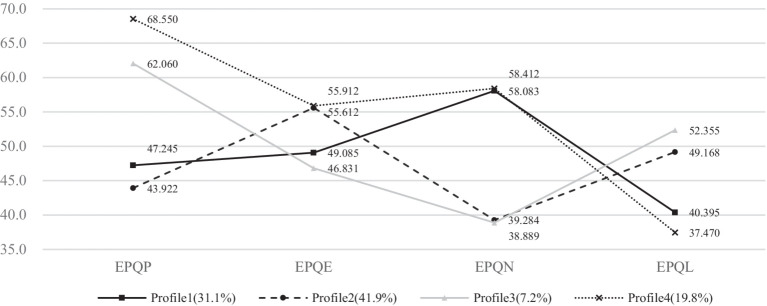
Four LPA modeling profiles.

- Profile 1 (31.1%): The Reserved Analyst. This profile is characterized by individuals who may be more prone to anxiety or worry (high neuroticism) but are less inclined toward impulsivity or aggression (low psychoticism), social engagement (low extraversion), and are less likely to manipulate the truth for self-presentation (low lie scores).
- Profile 2 (41.9%): The Social Diplomat. People in this group tend to be sociable and assertive (high extraversion) and may present themselves in a more favorable light (high lie scores), while being less prone to aggression or impulsivity (low psychoticism) and less susceptible to stress (low neuroticism).
- Profile 3 (7.2%): The Unconventional Pragmatist. These individuals tend to challenge the norm (high psychoticism), are not particularly open or engaging (low extraversion), are more emotionally stable (low neuroticism), and might be prone to dishonesty (high lie scores).
- Profile 4 (19.8%): The Impulsive Truth-Teller. This profile suggests individuals with intense emotions (very high neuroticism), a disregard for societal norms (very high psychoticism), an outgoing nature (very high extraversion), and a propensity to be honest (very low lie scores). It is important to note that the high psychoticism score in this profile does not necessarily imply a propensity for deception or a pervasive pattern of disregard for the rights of others, as seen in the DSM depiction of antisocial personality disorder. Instead, it suggests a tendency to question and challenge established norms and conventions, which may be perceived as unconventional or nonconformist behavior (Table 2 and Figure 3).

**Table 2:** Variance analysis of psychological scale scores across different profiles.

Scale	Profile 1	Profile 2	Profile 3	Profile 4	*F*	*p*	*η^2^*	*Post-test*
ERQ-CR	29.01 ± 4.705	30.29 ± 5.541	24.71 ± 3.838	24.86 ± 5.198	45.818	0.000	0.163	2 > 1 > 3, 2 > 1 > 4
ERQ-ER	16.69 ± 4.288	16.73 ± 4.461	16.55 ± 3.035	15.45 ± 3.299	3.486	0.016	0.015	1 > 4, 2 > 4
IUS-12	40.55 ± 6.784	36.90 ± 7.198	39.29 ± 4.571	37.70 ± 5.241	13.854	0.000	0.056	1 > 2, 1 > 4
MLQ	48.01 ± 8.081	52.11 ± 8.504	45.24 ± 7.140	48.65 ± 7.742	17.764	0.000	0.070	2 > 1, 2 > 3, 2 > 4
SCSQ-P	1.90 ± 0.443	2.09 ± 0.487	1.54 ± 0.522	1.60 ± 0.551	40.765	0.000	0.148	2 > 1 > 3, 2 > 1 > 4
SCSQ-N	1.34 ± 0.548	1.10 ± 0.490	1.24 ± 0.527	1.43 ± 0.514	16.831	0.000	0.067	1 > 2, 4 > 2
SIS	49.72 ± 5.777	54.83 ± 5.917	46.78 ± 3.449	47.43 ± 3.219	87.925	0.000	0.273	2 > 1 > 3, 2 > 1 > 4
SAI	47.07 ± 9.760	37.37 ± 9.689	49.82 ± 7.507	51.74 ± 7.686	99.130	0.000	0.297	4 > 1 > 2, 3 > 2, 4 > 2

### 3.3 Difference test

In the ANOVA conducted across the four distinct personality profiles identified through LPA on the EPQ, notable differences emerged, offering insights into the psychological dimensions of each profile. Profile 2, labeled ‘The Social Diplomat,’ demonstrates a robust mental health profile. This profile is marked by high scores in cognitive reappraisal (ERQ-CR), indicating an adeptness at emotionally adjusting to different situations through cognitive reframing. Additionally, members of this profile exhibit positive coping styles (SCSQ-P), suggesting effective strategies in managing stress and adversity. A notable finding is their high sense of life’s meaning (MLQ) and active social engagement (SIS), factors often associated with overall well-being and life satisfaction. These characteristics collectively suggest that Profile 2 may represent a relatively healthier psychological profile, particularly in aspects of emotional regulation, coping mechanisms, and social interactions.

Conversely, Profiles 1 and 4 exhibit traits that might correlate with certain psychological challenges. Profile 1, ‘The Reserved Analyst,’ shows a significant intolerance of uncertainty (IUS-12), potentially indicating a predisposition toward anxiety or stress in uncertain situations. This suggests a need for targeted interventions focusing on managing uncertainty and stress. Profile 4, ‘The Impulsive Truth-Teller,’ is characterized by low levels of expressive suppression (ERQ-ER), high levels of negative coping strategies (SCSQ-N), and high state anxiety (SAI). These traits imply a propensity for emotional volatility, difficulty in coping effectively with stress, and challenges in social interactions. Compared to Profile 2, these profiles may face more complex challenges in coping with stress, anxiety, and social engagement, highlighting the need for more specialized psychological support or interventions.

## 4 Discussion

This study aimed to analyze the relationships between Eysenck’s personality traits and a range of psychological constructs among university students, offering insights into their combined influence on students’ psychological well-being within the context of emerging adulthood. Using Latent Profile Analysis, we identified four distinct personality profiles: ‘The Reserved Analyst,’ ‘The Social Diplomat,’ ‘The Unconventional Pragmatist,’ and ‘The Impulsive Truth-Teller.’ These profiles exhibited significant differences in coping styles, anxiety levels, and other psychological aspects, highlighting the varied ways in which personality traits interact with psychological constructs in emerging adults.

The identification of distinct personality profiles among university students aligns with previous research emphasizing the heterogeneity of personality development during emerging adulthood (Schwartz et al., 2013). Our findings suggest that the unique challenges of this developmental stage, such as identity exploration and increased autonomy, may interact with personality traits to shape different patterns of psychological functioning (Arnett, 2023).

The ‘Social Diplomat’ profile, characterized by high extraversion and low neuroticism, appears to represent a relatively healthier psychological profile. This finding is consistent with studies showing that extraversion is associated with better social support and coping strategies in emerging adults (Roberts et al., 2006). Extraverted individuals may be better equipped to navigate the social challenges of university life, such as forming new relationships and seeking support when needed. Moreover, their lower levels of neuroticism may buffer against the stress and uncertainty that often characterize this developmental stage (Arnett, 2007).

Conversely, the ‘Reserved Analyst’ and ‘Impulsive Truth-Teller’ profiles, which exhibit traits like high neuroticism and low agreeableness, may be more vulnerable to the stressors of university life. These results align with research linking neuroticism to increased anxiety and depression in emerging adults (Kotov et al., 2010). Individuals with these profiles may struggle with the emotional and interpersonal demands of this stage, such as adapting to new living situations or dealing with academic pressures. Their lower levels of agreeableness may also hinder their ability to form supportive social networks, which are crucial for mental health during emerging adulthood (Barry and Madsen, 2010).

The ‘Unconventional Pragmatist’ profile, characterized by high psychoticism, presents an intriguing combination of traits that may be both adaptive and maladaptive in the context of emerging adulthood. On one hand, the creativity and non-conformity associated with psychoticism may facilitate the identity exploration and self-focus that are central to this stage (Klimstra et al., 2013). These individuals may be more willing to take risks and pursue unconventional paths, which can be important for personal growth. On the other hand, high psychoticism has also been linked to impulsivity and interpersonal difficulties, which may create challenges in the social and academic spheres of university life (Hengartner et al., 2018).

Our findings highlight the complex interplay between personality traits and the developmental tasks of emerging adulthood in shaping psychological well-being. The distinct personality profiles identified in this study suggest that university students may face different challenges and require tailored support based on their specific constellation of traits. For example, students with the ‘Reserved Analyst’ profile may benefit from interventions aimed at reducing anxiety and building social skills, while those with the ‘Social Diplomat’ profile may thrive with opportunities for leadership and peer support.

While our study found that the ‘Unconventional Pragmatist’ profile, characterized by high psychoticism, was associated with adaptive coping strategies, some previous research has linked psychoticism to maladaptive behaviors in emerging adults (Burt and Donnellan, 2008). This discrepancy may be due to differences in the specific facets of psychoticism assessed or the context in which these traits were examined. Further research is needed to clarify the role of psychoticism in the psychological functioning of emerging adults.

An intriguing finding of our study was the lack of significant differences in the presence of meaning in life across the personality profiles. This result suggests that the sense of purpose and meaning may be shaped by factors beyond personality traits in emerging adults, such as cultural influences or life experiences (Sharon, 2016). Future research could explore the interplay of personality and other contextual factors in shaping the search for meaning during this developmental stage.

Another unexpected finding was the similarity in emotion regulation strategies across the personality profiles, despite differences in anxiety levels. This result highlights the complexity of emotion regulation in emerging adulthood and suggests that factors beyond personality, such as social context or cognitive development, may play a role in shaping these processes (Gross, 2015). Further studies using more fine-grained measures of emotion regulation could provide a clearer picture of how personality interacts with these skills in emerging adults.

However, this study is not without limitations. The sample, restricted to university students from specific regions (Wenzhou and Nanjing), may not represent the broader population, given the unique experiences and psychological dynamics of university students compared to other groups (Rothman et al., 2013). Furthermore, the reliance on self-reported measures can introduce biases, such as social desirability or response bias (Gomes et al., 2019). Additionally, the cross-sectional nature of the study restricts our capacity to infer causality or observe the evolution of personality traits and psychological states over time.

The findings of this study have important implications for future research. First, future studies should further investigate the complex interplay between personality traits and psychological well-being among university students, exploring the relationships between personality profiles and other relevant factors such as academic performance, social support, and mental health help-seeking behaviors. Second, researchers should apply LPA to other student populations to examine the generalizability of our findings and investigate the effectiveness of tailored interventions designed to support students with specific personality profiles. These future directions will contribute to the development of evidence-based strategies to promote better mental health and well-being among university students.

## 5 Conclusion

This research provides an in-depth examination of personality profiles among university students within the frame of emerging adulthood, revealing the complex interplay between various personality traits and psychological constructs. Through the application of LPA to the EPQ and the correlation of these dimensions with other psychological measures, four distinct personality profiles were identified: ‘The Reserved Analyst,’ ‘The Social Diplomat,’ ‘The Unconventional Pragmatist,’ and ‘The Impulsive Truth-Teller.’ These profiles shed light on the intricate ways in which different personality traits combine to form unique psychological patterns.

## Data availability statement

The raw data supporting the conclusions of this article will be made available by the authors, without undue reservation.

## Ethics statement

The studies involving humans were approved by IRB in Wenzhou Seventh People’s Hospital. The studies were conducted in accordance with the local legislation and institutional requirements. The participants provided their written informed consent to participate in this study.

## Author contributions

L-LP: Writing – original draft. G-ZC: Writing – original draft, Data curation. S-RZ: Methodology, Writing – original draft. Y-DK: Data curation, Writing – review & editing. W-JY: Supervision, Writing – review & editing. Y-WW: Resources, Writing – review & editing. Z-YH: Resources, Writing – review & editing.
